# Successful Removal of Subretinal Silicone Oil Using a Vitreous Fluid Control Kit: A Case Report

**DOI:** 10.7759/cureus.99079

**Published:** 2025-12-12

**Authors:** Tadanobu Sato, Ayako Saito

**Affiliations:** 1 Department of Ophthalmology, Uonuma Kikan Hospital, Minami Uonuma, JPN

**Keywords:** pars plana vitrectomy, retinotomy, rhegmatogenous retinal detachment, subretinal silicon oil, vitreous fluid control

## Abstract

Silicone oil (SO) is commonly used as a long-term intraocular tamponade in complex retinal detachment cases, but its use may lead to several complications, including glaucoma, keratopathy, cataract, and oil emulsification. Subretinal migration of SO is an uncommon but clinically significant event that can occur through pre-existing or iatrogenic retinal breaks or microscopic defects caused by repeated intraocular manipulation. We report a rare case of subretinal SO migration following pars plana vitrectomy (PPV) that was successfully managed using a direct intraocular approach with a vitreous fluid control (VFC) kit. A 48-year-old male patient presented with decreased vision in the left eye. Fundus examination revealed total rhegmatogenous retinal detachment with proliferative vitreoretinopathy. Initial surgery involved scleral buckling and sulfur hexafluoride gas injection. Due to persistent subretinal fluid, a second PPV was performed with posterior vitreous detachment induction, removal of subretinal bands, PFCL injection, and SO tamponade. Seven days postoperatively, subretinal migration of SO was observed. A third PPV was performed to remove subretinal SO. A retinal incision was created at a location that allowed straight access via a nasal trocar. The 25G cutter failed to aspirate the SO; however, the VFC kit enabled successful removal. The retina remained attached postoperatively. SO removal and intraocular lens fixation were later performed without recurrence. This case demonstrates that strategic intraocular access using VFC-assisted aspiration can enable effective removal of subretinal SO while minimizing surgical damage and emphasizes the importance of careful planning during vitreoretinal surgery.

## Introduction

Silicone oil (SO) is widely used as a long-term intraocular tamponade agent for retinal reattachment, particularly in complex cases such as proliferative vitreoretinopathy (PVR), giant retinal tears, or severe traumatic retinal detachment. Its chemical stability, optical transparency, and buoyancy make it a valuable material in vitreoretinal surgery. However, the use of SO is not without complications. Well-documented adverse effects include secondary glaucoma, corneal decompensation, cataract formation, and emulsification leading to anterior chamber migration [1,2]. In addition to these well-known complications, long-term retention of SO has been reported to induce structural and functional changes in the retina. Experimental and clinical studies have shown that prolonged contact between SO and retinal tissue may lead to degeneration of photoreceptor cells and Müller cell activation, suggesting a direct toxic effect of SO on retinal architecture and visual function [3,4]. Although less common, the migration of SO into the subretinal space is a rare but clinically significant event. The mechanisms of subretinal SO migration include the presence of undetected retinal breaks, reopening of pre-existing holes, or iatrogenic defects created during fluid-air or air-SO exchange. In addition, repeated surgical manipulation, intraoperative use of perfluorocarbon liquid (PFCL), and emulsified oil droplets can contribute to the passage of SO through the retina into the subretinal space [5-7]. Subretinal migration of SO can impair photoreceptor function, cause localized retinal toxicity, and make further retinal reattachment challenging [3,4]. Moreover, surgical removal of subretinal SO is technically demanding due to the limited access and potential for iatrogenic retinal damage. Herein, we report a rare case of subretinal SO migration following pars plana vitrectomy (PPV), which was successfully removed using a direct intraocular approach with minimal retinotomy.

## Case presentation

A 48-year-old man who had been experiencing a gradual loss of vision in his left eye for about five years was examined at a local doctor's clinic and was referred to our hospital because he was found to have retinal detachment with a subretinal strand.

**Figure 1:**
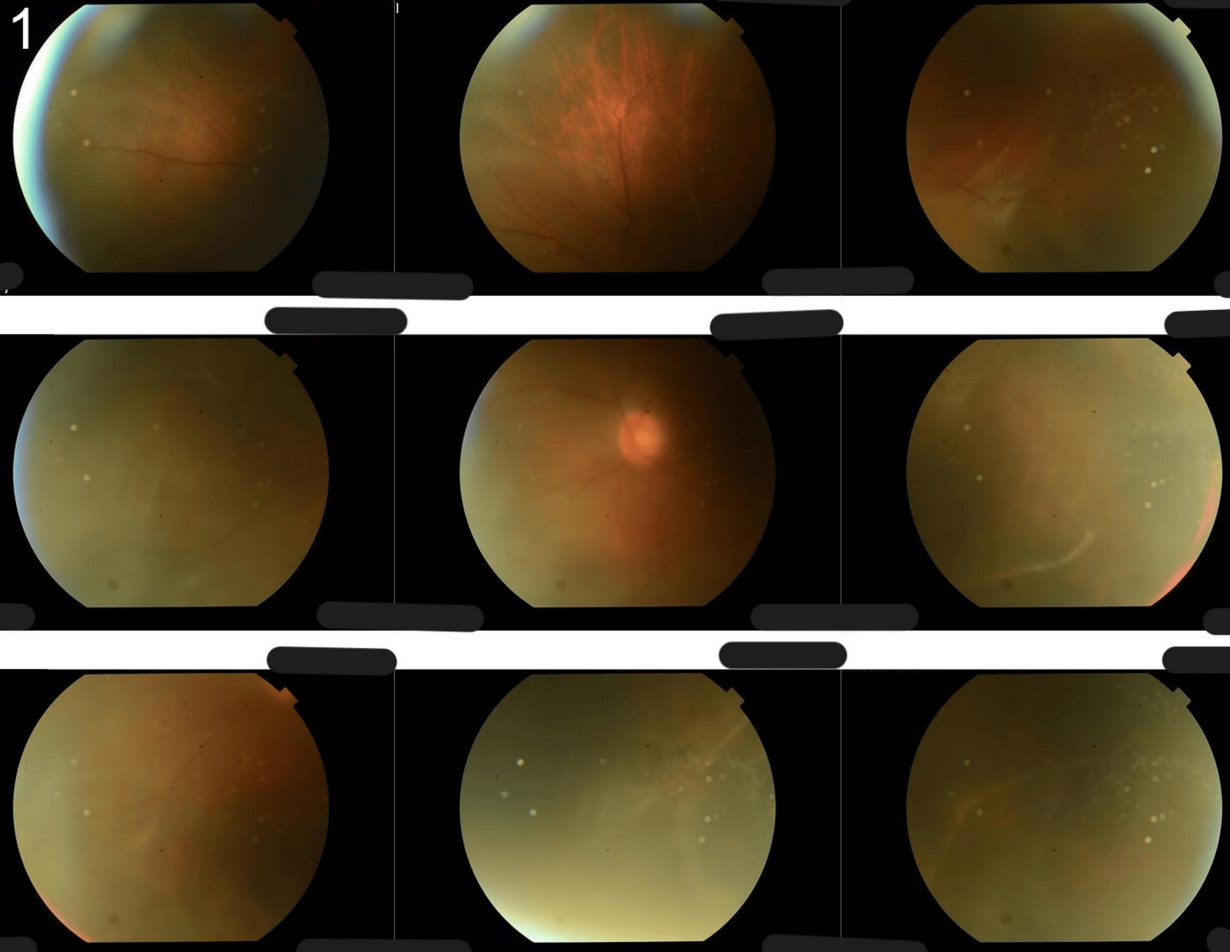
Fundus photographs at the initial visit showing total retinal detachment Wide-field fundus photographs taken at the initial presentation. A total retinal detachment involving the macula is observed. No obvious retinal breaks, tears, or atrophic holes are identifiable.

At our examination, His best-corrected visual acuity (BCVA) (logMAR) was -0.08 in his right eye and 2.00 in his left eye. He had intraocular pressures (IOP) of 22 mmHg in the right eye and 9 mmHg in the left eye. During the slit-lamp examination, silicone intraocular lenses were observed in both eyes, and there was a retinal detachment and subretinal fibrosis in the left eye. Although a small circular hole in the lower peripheral area of the left eye was identified intraoperatively, it was not detectable in the preoperative fundus photographs. There were no abnormal findings in the anterior chamber, and there was no posterior vitreous detachment (Figure 1). He had atopic dermatitis as a previous medical condition.

An encircling scleral buckling was performed under general anesthesia 11 days after the initial visit. The surgery was performed as follows: the conjunctiva was incised circumferentially, and traction threads were passed through the superior rectus muscle, medial rectus muscle, inferior rectus muscle, and external rectus muscle. A small atrophic hole was observed in the lower region. The subretinal fluid was drained through the scleral wound, and cryoretinopexy was performed on the inferior circular hole and the area of degeneration. Next, a silicone tire was placed around the circumference and fixed with 5-0 polyester suture, and 100% sulfur hexafluoride 0.6 ml was injected into the eye.

Because the subretinal fluid gradually increased after the surgery and the retina could not be repositioned, PPV was performed about two months after the encircling scleral buckling. The PPV was performed as follows: PPV was performed under sub-tenon anesthesia, followed by core and peripheral vitrectomy, Removal of proliferative membrane and retinal fibers around the hole that caused the retinal detachment, perfluorocarbon liquid (PFCL) injection, subretinal fluid drainage, fluid-air exchange, retinal photocoagulation, and air-SO exchange.

**Figure 2:**
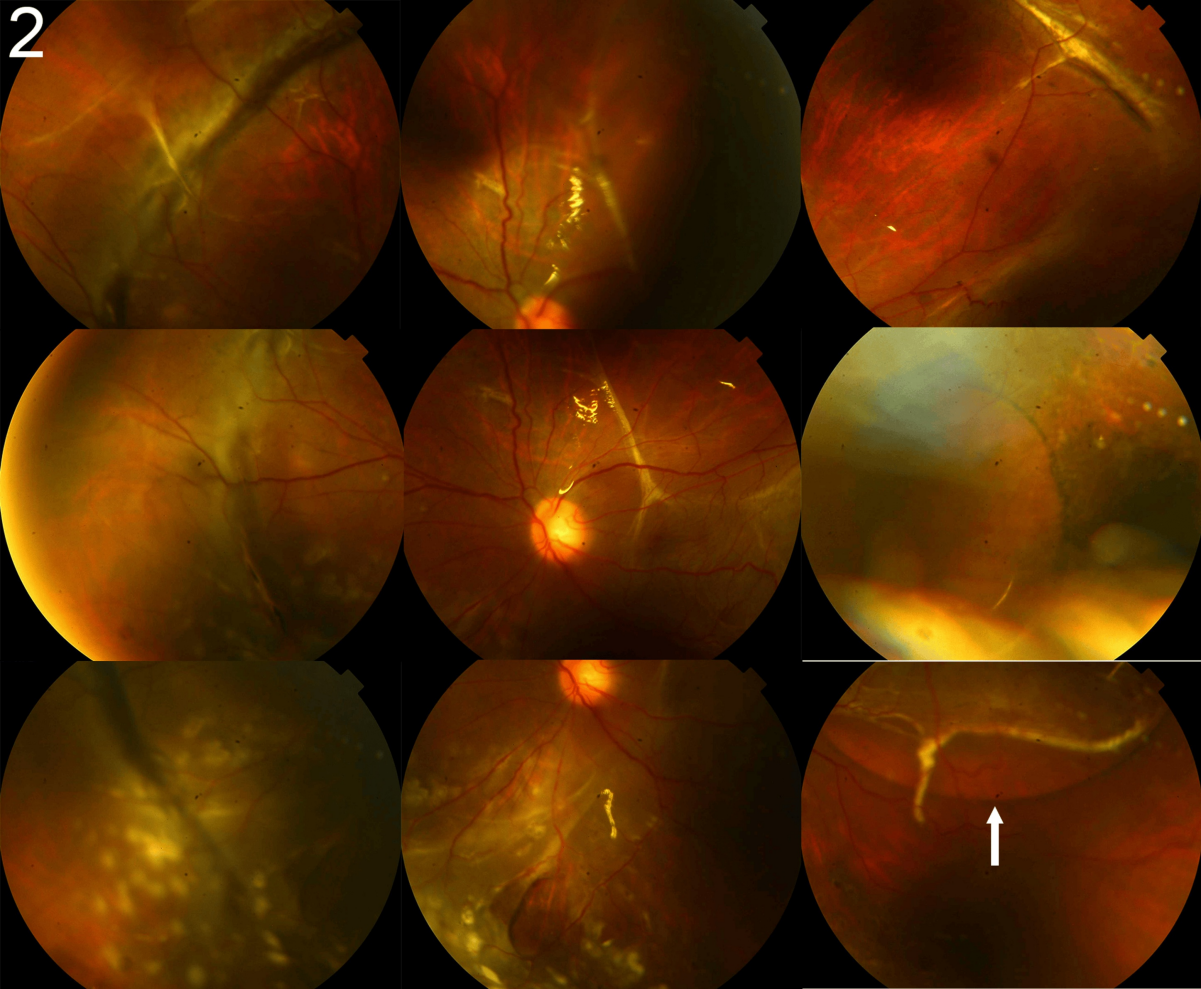
Subretinal silicone oil observed after the initial pars plana vitrectomy Fundus photographs showing subretinal silicone oil bubbles following the initial pars plana vitrectomy. The subretinal silicone oil (white arrow) appears as round, well-defined, spherical bubbles that are clearly separated from the surrounding subretinal fluid.

Following the second surgery, subretinal fluid gradually increased from a lower retinal tear, and a spherical substance suspected to be SO persisted (Figure 2). Therefore, PPV was performed on the seventh postoperative day.

**Figure 3:**
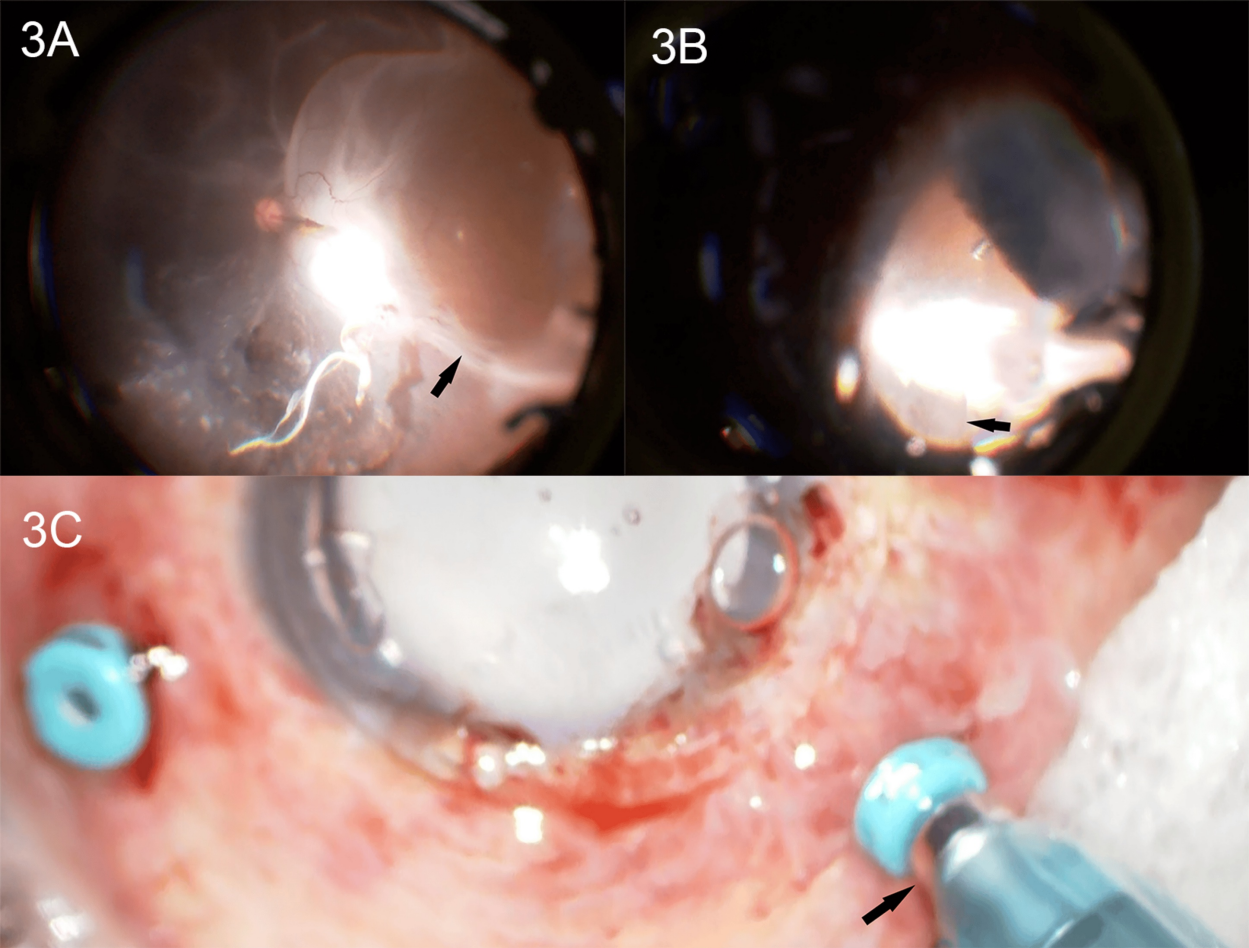
Intraoperative findings during removal of subretinal silicone oil (A) Intraoperative fundus photograph showing a large subretinal silicone oil bubble (black arrow) before drainage. (B) Release of subretinal silicone oil (black arrow) observed after creating a small drainage retinotomy. (C) External surgical view showing silicone oil removal using a vitreous fluid control kit (black arrow).

A retinotomy approximately the size of the optic disc was created. The incision site was chosen to facilitate the removal of the subretinal cord-like material and to allow a straight-line approach from the position of the superior nasal trocar. The 25G vitreous cutter could not aspirate the SO; aspiration was successfully performed using the VFC kit (Figures 3A-3C). The surgeon moved appropriately between the cephalic and temporal sides to remove the subretinal strands. PFCL was injected, followed by fluid-air exchange and filling with SO, after which the incision was closed.

**Figure 4:**
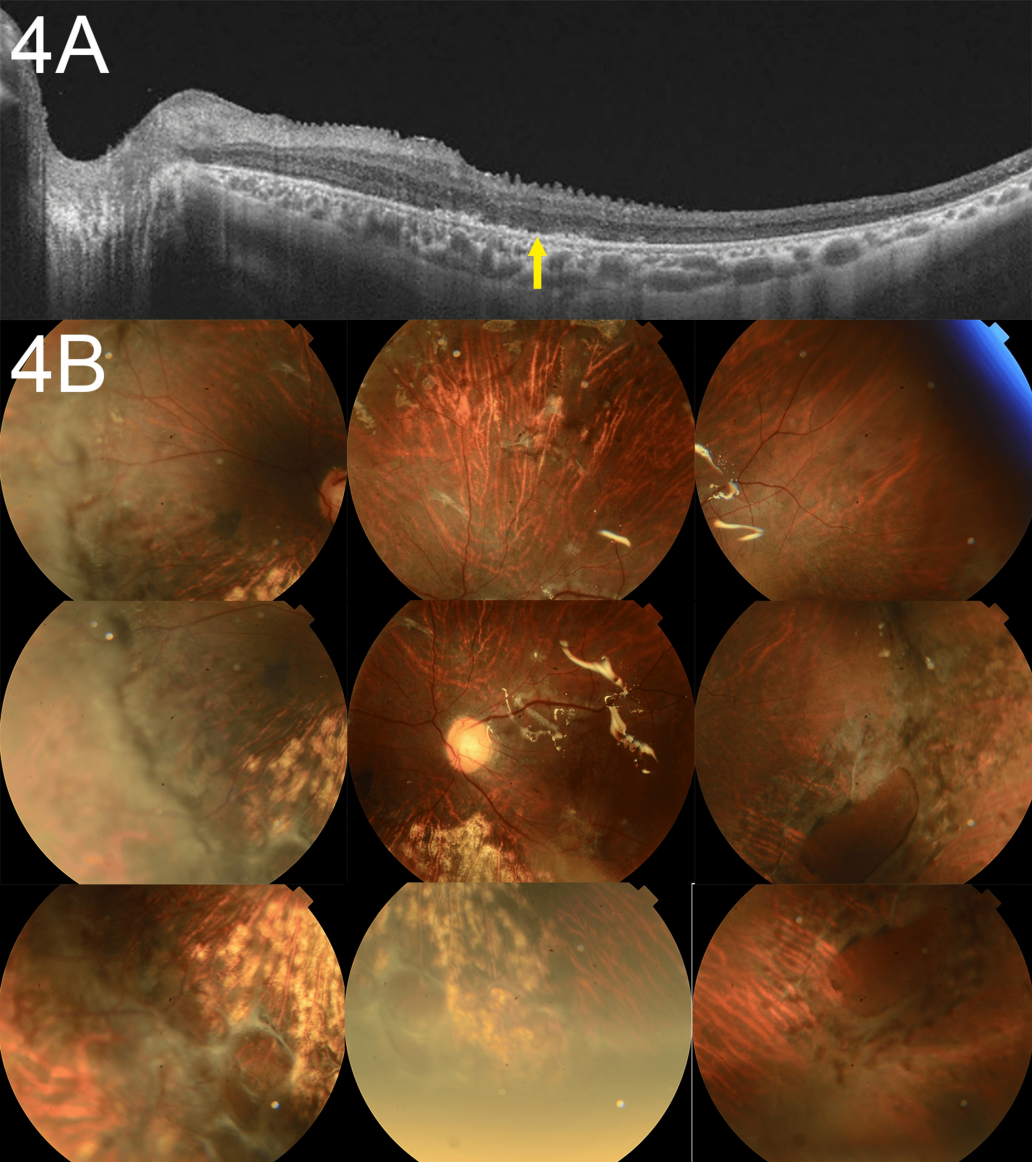
Postoperative imaging showing complete retinal reattachment at three months after surgery (A) Postoperative optical coherence tomography image of the macula three months after surgery, showing complete retinal reattachment. Outer retinal layer defects, including ellipsoid zone and external limiting membrane disruptions, are still visible (yellow arrow). (B) Fundus photographs of the right eye three months after silicone oil removal, demonstrating complete retinal reattachment and resolution of the previously retained subretinal silicone oil bubbles. The retinal surface appears stable without signs of recurrent detachment or proliferative vitreoretinopathy.

At 3 months postoperatively, the left eye had a BCVA (logMAR) was 1.30 with an IOP of 25 mmHg. There was no corneal damage, and retinal reattachment was achieved (Figures 4A, 4B). At 6 months postoperatively, the SO was removed, and an intraocular lens was scleral-fixed. The patient has progressed without re-detachment.

## Discussion

Subretinal migration of SO is a rare but clinically significant complication following vitreoretinal surgery. A large nationwide survey in Japan reported that subretinal migration occurred in only 31 of 2,170 cases (1.4%) in which SO was used as an intraocular tamponade [8]. The actual incidence may be underestimated, as small bubbles can remain undetected on fundus examination or OCT. Although several mechanisms of subretinal SO migration have been described, in our case, the intraoperative findings suggested that the silicone oil entered the subretinal space through the inferior circular retinal break.

Several previous studies have described similar cases of subretinal silicone oil and its characteristic findings on imaging. Linton and Jasani reported that subretinal SO can appear as a dome-shaped, hyporeflective lesion with choroidal shadowing on OCT, which may mimic retained perfluorocarbon liquid [5]. Chitolina and Bacin also reported successful removal of a subretinal SO bubble through an internal drainage approach using a retinotomy, highlighting the technical difficulty of removing SO without causing additional retinal injury [9].

Once SO migrates into the subretinal space, it is usually difficult to remove through a small retinotomy, and a relatively large retinotomy is often required to ensure adequate evacuation of the SO [9]. Alternative approaches, such as external drainage through the sclera, have also been described, similar to subretinal fluid drainage performed during scleral buckling surgery [10]. However, these methods are technically demanding and carry risks of choroidal hemorrhage or retinal incarceration. In this case, we considered but did not perform the removal of subretinal SO using PFCL. This was because we believed the method is effective only when SO is mobile and not enclosed by subretinal fibrous strands.

In the present case, external drainage was not feasible because the patient had previously undergone scleral buckling. Although the use of PFCL to displace the subretinal SO was initially considered, dense subretinal fibrous strands were thought to prevent adequate mobilization of the SO. Therefore, a direct intraocular approach was chosen. By aligning the new retinotomy along the same axis as the nasal-superior trocar and the subretinal SO bubble, we were able to use the VFC kit effectively to aspirate the SO. This approach minimized additional retinotomy while allowing complete removal of the subretinal SO.

Previous studies have suggested that retained or long-standing SO can induce localized retinal toxicity and impair photoreceptor function. Karaca et al. demonstrated thinning of the outer retinal layers and limited visual recovery in eyes with prolonged SO tamponade [4]. In addition, Pichi et al. reported inner retinal changes consistent with silicone oil-related toxicity, reinforcing the need for early removal of SO when possible to prevent further functional decline [3]. Therefore, prompt removal of subretinal SO, as in this case, is also important to minimize the risk of retinal toxicity.

Postoperatively, the retina remained attached without recurrence of detachment. Although the IOP was slightly elevated, there were no corneal complications, and a stable retinal configuration was achieved after subsequent SO removal and secondary intraocular lens fixation. This case demonstrates that subretinal SO can be successfully removed via a controlled intraocular approach when careful planning of the retinotomy site and the surgical trajectory is performed. Moreover, the case underscores the importance of identifying the possible routes of SO migration, minimizing unnecessary retinal manipulations, and ensuring watertight retinal sealing during the initial procedure. Meticulous intraoperative inspection of the peripheral retina and judicious management of subretinal strands may help prevent this rare but challenging complication.

Postoperatively, the retina remained attached without recurrence of detachment. Although the IOP was slightly elevated, there were no corneal complications, and a stable retinal configuration was achieved after subsequent SO removal and secondary intraocular lens fixation. This case demonstrates that subretinal SO can be successfully removed via a controlled intraocular approach when careful planning of the retinotomy site and the surgical trajectory is performed. Moreover, the case underscores the importance of identifying the possible routes of SO migration, minimizing unnecessary retinal manipulations, and ensuring watertight retinal sealing during the initial procedure. Meticulous intraoperative inspection of the peripheral retina and judicious management of subretinal strands may help prevent this rare but challenging complication.

## Conclusions

Subretinal migration of SO is a rare but clinically significant complication that may require innovative intraocular techniques for effective management. The present case highlights the importance of careful surgical planning and the usefulness of VFC-assisted aspiration in achieving successful anatomical reattachment with minimal retinotomy. Early identification and prompt removal of subretinal SO are essential to prevent potential retinal toxicity and optimize visual outcomes. Furthermore, this case underscores the need for meticulous intraoperative inspection of the peripheral retina and careful handling of subretinal strands to minimize the risk of this uncommon yet challenging complication.

## References

[1] Abu-Yaghi NE, Abu Gharbieh YA, Al-Amer AM (2020). Characteristics, fates and complications of long-term silicone oil tamponade after pars plana vitrectomy. BMC Ophthalmol.

[2] Łątkowska M, Gajdzis M, Kaczmarek R (2024). Emulsification of silicone oils: altering factors and possible complications-a narrative review. J Clin Med.

[3] Pichi F, Hay S, Abboud EB (2020). Inner retinal toxicity due to silicone oil: a case series and review of the literature. Int Ophthalmol.

[4] Karaca U, Kucukevcilioglu M, Durukan AH, Akincioglu D (2022). The effect of longstanding silicone oil on retina choroid and optic nerve in eyes with retinal detachment: an optical coherence tomography study. BMC Ophthalmol.

[5] Linton E, Jasani K (2023). Subretinal silicone oil: OCT imaging findings and surgical management of a rare and unusual iatrogenic complication of retinal detachment surgery. BMJ Case Rep.

[6] Fukumoto M, Nishida Y, Kida T, Sato T, Kobayashi T, Ikeda T (2018). A case of silicone oil adhered to the retinal surface via perfluorocarbon liquid. BMC Ophthalmol.

[7] Valentín-Bravo FJ, Stanga PE, Reinstein UI, Stanga SE, Martínez-Tapia SA, Pastor-Idoate S (2024). Silicone oil emulsification: a literature review and role of widefield imaging and ultra-widefield imaging with navigated central and peripheral optical coherence tomography technology. Saudi J Ophthalmol.

[8] Sakamoto T, Hida T, Tano Y (2008). Survey of silicone oil for ocular diseases in Japan [Article in Japanese]. Nippon Ganka Gakkai Zasshi.

[9] Chitolina J, Bacin F (2006). Treatment of a subretinal silicone oil bubble. J Fr Ophtalmol.

[10] Shanmugam MP, Ramanjulu R, Kumar RM, Minija CK (2012). Transscleral drainage of subretinal/suprachoroidal silicone oil. Ophthalmic Surg Lasers Imaging.

